# Pupillary responses to directional uncertainty while intercepting a moving target

**DOI:** 10.1098/rsos.240606

**Published:** 2024-10-02

**Authors:** Inmaculada Márquez, Mario Treviño

**Affiliations:** ^1^ Departamento de Ciencias Médicas y de la Vida, Centro Universitario de la Ciénega, Universidad de Guadalajara, Ocotlán, Mexico; ^2^ Laboratorio de Conducta Animal, Departamento de Psicología, Centro Universitario de la Ciénega, Universidad de Guadalajara, Ocotlán, Mexico; ^3^ Laboratorio de Plasticidad Cortical y Aprendizaje Perceptual, Instituto de Neurociencias, Universidad de Guadalajara, Guadalajara, Jalisco, Mexico

**Keywords:** pupils, uncertainty, foveal vision, interception, predictive processing

## Abstract

Pupillary responses serve as sensitive indicators of cognitive processes, attentional shifts and decision-making dynamics. Our study investigates how directional uncertainty and target speed (*V*_T_) influence pupillary responses in a foveal tracking task involving the interception of a moving dot. Directional uncertainty, reflecting the unpredictability of the target’s direction changes, was manipulated by altering the angular range (AR) from which random directions for the moving dot were extracted. Higher AR values were associated with reduced pupillary diameters, indicating that heightened uncertainty led to smaller pupil sizes. Additionally, an inverse U-shaped relationship between *V*_T_ and pupillary responses suggested maximal diameters at intermediate speeds. Analysis of saccade-triggered responses showed a negative correlation between pupil diameter and directional uncertainty. Dynamic linear modelling revealed the influence of past successful collisions and other behavioural parameters on pupillary responses, emphasizing the intricate interaction between task variables and cognitive processing. Our results highlight the dynamic interplay between the directional uncertainty of a single moving target, *V*_T_ and pupillary responses, with implications for understanding attentional mechanisms, decision-making processes and potential applications in emerging technologies.

## 1. Introduction

Eye movements are crucial for exploring the visual world and require multiple brain areas, including the frontal and parietal eye fields, superior colliculus, cerebellum and brainstem [1]. Additionally, regulating pupillary responses is integral to the visual system, enhancing our ability to perceive and respond to variations in luminance within our visual environment. Dilation, controlled by the sympathetic nervous system, enlarges the pupils in response to low-light conditions. Conversely, under parasympathetic control, constriction shrinks the pupils in bright light and during close-up visual tasks [1,2]. The human pupil typically measures around 3 mm on average, but it can vary from 2 to 8 mm in response to different light intensities [3,4]. Changes in pupillary size also exist in stable luminance conditions but are more modest, rarely exceeding 0.5 mm [5,6]. These variations in pupil diameter occur in both eyes and are not subject to conscious control [5,7]. Moreover, task-specific cognitive demands can cause noticeable changes in pupillary responses. Interestingly, a link between pupil size and cognitive–emotional states has been suggested for a long time. For instance, researchers have proposed that pupillary responses reflect various functions, including changes in central arousal, motivation, prediction of reward and error-making [2,8]. Hence, pupillary responses contain useful information about cognitive functions, including changes in attention and decision-making.

Stimuli inducing arousal, curiosity and surprise can trigger pupil dilation, occurring simultaneously with internally generated cognitive events such as mental calculations and decisions [9]. However, linking pupillary sizes to specific perceptual and cognitive functions faces challenges due to the slow dynamics of pupillary responses. Pupil size is controlled by two muscle sets: the circular sphincter pupillae contract to reduce pupil size, while the radial dilator pupillae contract to widen them. These smooth muscle fibres operate much slower than skeletal muscles (~100 times slower), gradually changing pupil size [10]. Yet, despite their slower dynamics, pupil diameters are valuable for probing continuous and anticipatory processing across experimental trials lasting several seconds, with the potential for tracking task-specific cognitive events [5].

Contemporary theories posit pupil size as the output of a dilatory low-pass filter synchronized with task-related events [11–14]. Oculomotor and pupillary responses, including dilation and constriction, work together via shared neural pathways to enhance visual perception and adapt to task changes [15,16]. Saccades are rapid eye movements redirecting focus between visual field areas, and are closely associated with changes in pupil size [1,4]. This relationship is influenced by task complexity and perceptual demands, with saccades often causing pupil constriction followed by dilation. During saccades, the eye rapidly shifts to bring a new visual field onto the fovea, a retinal region characterized by its high concentration of photoreceptors and sharpest visual acuity [17]. Thus, central vision exhibits a greater density of cones and one-to-one connections between photoreceptors and ganglion cells, facilitating detailed vision. In contrast, peripheral vision features lower cone density and shared ganglion cells, leading to reduced acuity [18]. With saccades, rapid movements induce fluctuations in incoming light, impacting pupil size. Despite controlling for light, pupil size correlates strongly with saccadic movements across tasks, showing a pattern of constriction followed by dilation synchronized with saccades [15,16]. Therefore, changes in pupil size, correlated with oculomotor actions like saccades and blinks, may reflect cognitive effort in saccade planning and execution. Interconnected brain circuits, involving various regions and neural pathways, orchestrate oculomotor movements and pupil responses, optimizing visual perception. This relationship extends to saccade response times and inhibition, influenced by task complexity and perceptual detection [6]. The superior colliculus (SC), particularly its intermediate layers (SCi), regulates saccades and influences pupil dilation responses [19,20]. Exploring the interplay between saccades and pupil responses is crucial, as saccades may affect pupil diameter through changes in cognitive effort, especially in tasks of varying difficulty [21]. Different forms of uncertainty have been shown to affect cognitive load and decision-making processes [22,23], which in turn can influence pupillary responses.

Some studies suggest uncertainty demands cognitive effort, altering pupillary diameters [24,25]. Several forms of uncertainty have been classified in decision-making studies [22,26]. Expected uncertainty occurs when the probability of outcomes from learned contingencies is known but unreliable, allowing agents to predict outcomes based on past experiences. Unexpected uncertainty arises from abrupt, fundamental changes in the environment that disrupt previously established contingencies, challenging the agents' ability to predict outcomes based on historical data. Volatility introduces another dimension, characterized by frequent and unpredictable changes in contingencies over time. This form of uncertainty demands continuous adaptation and updating of agents' understanding of the environment to maintain accurate predictions and effective decision-making. Thus, for example, in a motion coherence task using peripheral vision, pupillary responses were smaller after accurate decisions with low motion uncertainty but larger after errors [27]. Both expected and unexpected forms of uncertainty have been associated with pupil responses, suggesting brain arousal during the updating of models following environmental changes. Other studies have suggested that pupillary dilation mirrors human inferences on environmental volatility [28], and variations in pupil diameter during steady gaze show sensitivity to task difficulty [29]. Therefore, although a few studies have found no significant link between pupil size and uncertainty [30], potentially influenced by individual differences or task-specific variations, the overall evidence suggests a potential connection between pupillary responses and uncertainty processing. This relationship, however, remains unknown in tasks requiring focussed, foveal vision. Previous studies have primarily focused on static visual tasks without ocular movements, leading to limited knowledge of how directional uncertainty influences pupillary responses during continuous and interactive tasks. While saccadic movements and their associated pupillary changes are well-documented in static contexts, their interaction with uncertainty in dynamic environments remains largely unexplored. Moreover, the specific interaction between saccade-triggered pupillary responses and varying levels of directional uncertainty has not been investigated.

Our study examined how directional uncertainty in a task requiring continuous visual and motor engagement affects average and saccade-triggered pupillary responses. By manipulating the angular range (AR) and target speed (*V*_T_) of a moving target, we explored the pupillary dynamics during interception. Our results contribute to a broader understanding of the neural mechanisms underlying attention, perception, and motor control, informing theoretical models of cognitive processing and supporting the development of more accurate simulations of human behaviour. Additionally, insights into how uncertainty affects cognitive load and performance can have practical implications for designing training programs in fields such as aviation, sports and military operations, where individuals often operate under uncertain and dynamic conditions.

## 2. Material and methods

### 2.1. Participants

We conducted experiments with 50 right-handed adults, comprising 25 women and 25 men, ages 18–29 years (mode of 21). All participants in our study were enrolled at the Universidad de Guadalajara, Mexico’s second-largest public university. The institution attracts students from a wide range of socioeconomic backgrounds, resulting in a sample that includes individuals from various socioeconomic levels. This diversity enhances the potential for generalizing our findings within this context. However, our study’s focus on a single geographic location would require further research to determine the applicability of our results to other populations and settings. Participants were assigned to one of two primary experiments. In the first experiment (E1), involving nine hundred trials, participants experienced specific variations in the directional uncertainty of the moving target by adjusting the AR (see description in the following text), with four main groups experiencing different changes in AR throughout the trials. The second experiment (E2) comprised six hundred trials and involved sinusoidal variations of AR, *V*_T_ or a combination of both (six full cycles). We established clear criteria for participant inclusion, requiring no reported prenatal, perinatal, or postnatal issues that could impact nervous system development. None of the participants had been diagnosed with psychiatric, neurological or neurodevelopmental disorders, nor did they have a history of substance use. All possessed normal or corrected-to-normal vision, as assessed by the Snellen test. In alignment with ethical guidelines and research standards, we collected demographic data from participants to ensure a reasonable description of our study sample. The dataset encompassed demographic information such as age, gender and socioeconomic status, collected via self-reported questionnaires. Emphasizing privacy and confidentiality, participants were assured that their responses would remain strictly confidential. Participants were provided written instructions for completing questionnaires and performing the task. Participation was entirely voluntary and did not involve any financial incentives, with participants retaining the autonomy to withdraw or discontinue their involvement in the experiments at any time. All procedures adhered to non-invasive protocols, complied with local guidelines and regulations, and received approval from the Instituto de Neurociencias ethics committee, Universidad de Guadalajara, México, under the reference ET102021-330.

### 2.2. Visuomotor task

Participants sat in front of a computer screen and engaged in a simple visuomotor task where they chased a computer-controlled dot [21]. The task was displayed on a 27-in. computer monitor (1920 × 1080 pixels, 60 Hz), with two small dots (each 0.3° in visual angle) representing the participant (white dot) and the moving computer-controlled target (black dot). These dots were presented within a circular arena against a 50% grey background. Participants controlled the white dot using a joystick with their dominant hand (Logitech Extreme three-dimensional Pro). This joystick is primarily designed for right-handed users, with an ergonomic shape and button placement optimized for right-hand control. Although we did not conduct specific experiments on left-handed individuals, we do not anticipate performance differences if a left-handed joystick had been available. The joystick was calibrated daily, and we tracked the joystick’s position on the monitor via a custom-made program. Each trial started with the participant’s dot at the screen’s centre, and the computer-controlled target appeared randomly along the circular arena’s edges. The objective was to chase the moving target, and trials ended when the dots collided (collision trial) or if the trial duration exceeded 5 s (no-collision trial). Auditory feedback (pure tones) indicated trial success or failure [31]. We quantified collision time (CT) as the duration from the trial initiation, where dots were projected onto the screen, to the moment of collision (shorter CTs indicated better task performance). After each trial, participants were required to return the joystick to its initial centred position before starting the subsequent trial. We adjusted task difficulty by manipulating two parameters. We introduced variability in the target’s direction by employing variable AR, from which random directions were selected (in E1). We also introduced variability in AR, *V*_T_ or both, which changed sinusoidally across trials (in E2). Higher *V*_T_ increased the difficulty of intercepting the target [21]. If the target reached the edges of the circular arena, we applied the law of reflection, which involved adjusting the angle of the target’s path by adding the specified angular variability for each trial. Participants were instructed to remain still and minimize head movements while allowing gaze adjustments during the experiment. We limited each session to a maximum of 900 trials to ensure active participation and optimal task performance, incorporating two 5 min resting periods [31,32]. Experimental sessions typically lasted less than an hour. The visuomotor task was programmed using MATLAB R2023a and implemented with the Psychophysics Toolbox extensions (PTB-3).

### 2.3. Gaze-pupil tracking

We utilized a high-precision commercial eye tracker (Tobii, Stockholm, Sweden; sample rate: 60 Hz) to investigate the oculomotor and pupillary responses associated with successful interception. The eye tracker was positioned at the lower part of the monitor, and participants were seated at a 70 cm distance from the screen (measured along a diagonal) to maintain consistency. We employed a chin holder with a forehead rest to minimize head movements (i.e. head-stabilized recordings). We maintained stable room lighting at approx. 100 lux, measured at a distance of 70 cm from the monitor, and used curtains to ensure consistent lighting conditions for precise pupillary measurements [33]. The eye tracker with stereo cameras, capturing images from both eyes, recorded gaze positions and pupillary diameters. Because precise ocular measurements depend on proper calibration, we first ensured the visibility of pertinent eye features, including the pupil and corneal reflections, before applying the following calibration routines to each participant [2]. The initial calibration employed a conventional four-point procedure directly from the eye tracker’s software, with participants fixating on screen corners. Subsequently, we performed a MATLAB-based calibration utilizing the Psychophysics Toolbox as the second routine. Participants fixated on four white dots with a 0.6° outer diameter around the circular arena’s periphery. This step helped us confirm the proper alignment of the eye-tracking data with the visual stimuli projection. The third calibration routine tested the tracking accuracy for smooth-pursuit eye movements [1,4]. During this routine, we recorded the participants' gaze position and velocity using the eye tracker and compared them with the position and velocity of a moving dot. The collected data were subsequently employed to calculate participants' smooth-pursuit gain and measure the gaze velocity to dot velocity ratio [4]. In the fourth routine, we collected pupillary data during free eye viewing and saccade production. We recorded ocular movements as participants freely looked around a blank arena (i.e. without the participant and computer dots). The instructions here encouraged participants to perform saccadic movements actively, enabling rapid changes in their gaze positions within the arena. This procedure helped us establish a baseline reference for the saccade-triggered pupillary changes that occur without visual stimuli. The fifth and final calibration routine elicited the pupillary light reflex. During each trial, lasting approx. 35 s, participants experienced alternating contrast conditions between black-and-white screens (six full cycles), with this sequence repeated four times.

### 2.4. Analysis

Gaze and pupillary traces were imported into MATLAB. As a first method to preprocess pupillary responses, we used a percentile-based divisive standardization which used specific percentile values (5th and 95th) from the pupillary light reflex response to create a normalized scale. Specifically, we standardized the observed pupillary diameters for each trial from each participant by subtracting the 5th percentile of the pupillary light reflex response (data from six full contrast cycles repeated four times) and then dividing the result by the amplitude response, which was calculated as the difference between the 95th and 5th percentiles of the pupillary light reflex obtained during the fifth calibration routine. Electronic supplementary material, figure S2, illustrates the stability of these measures, showing consistent percentile values before and after the experiments. To validate this procedure, we examined a subset of 31 participants from E1. First, we assessed the reliability of the eye-tracking system by repeating the four-point fixation calibration routine five minutes after participants finished their experimental sessions. Horizontal and vertical fixations were consistent (paired *t*‐test, horizontal fixations: *p* = 0.71; vertical fixations: *p* = 0.26; ANOVA test with Bonferroni post hoc test, horizontal fixations: *F*_1,59_ = 0.01, *p* = 0.91; vertical fixations: *F*_1,59_ = 0.45, *p* = 0.33, *n* = 31 participants from E1, electronic supplementary material, figure S2a). Next, we assessed the reliability of pupillary response measurements by repeating the fifth calibration routine at the end of the experiments. We calculated the area under the curve (AUC) for normalized pupillary diameters aligned to the moment of collision before the trial concluded. This metric integrates dynamic changes in pupillary responses, minimizing noise impact and facilitating data interpretation. Comparisons of the AUC, 5th and 95th percentiles, and average pupillary responses before and after the experiments showed no significant differences (paired *t*‐test, AUC: *p* = 0.88, not illustrated; 5th percentile: *p* = 0.87; 95th percentile: *p* = 0.11; Av. Pupillary response: *p* = 0.33, *n* = 31; electronic supplementary material, figure S2bc). These results confirm the reliability of our measurements, allowing precise measurements and consistent comparisons. As a second method, we used a subtractive approach to standardize pupillary responses by subtracting the mean pupil size from all subsequent pupil-size measurements, ensuring the pupil size starts from 0 during the baseline period on every trial [34].

To search for potential pupillary differences between collision and no-collision trials, we aligned pupillary responses to either the moment of collision (for collision trials) or the end of the trial (for no-collision trials, 5-s trials). However, the inherent difference in trial durations between these two conditions posed a challenge for a direct comparison. To address this problem, we implemented a method to match the trial durations across conditions. Therefore, pupillary response traces from no-collision trials were systematically clipped and adjusted to match the duration of collision trials. This involved randomly selecting each participant’s trial durations from collision trials (with no replacement). We refer to the resulting clipped traces as pupillary responses from ‘no-collision trials with matched duration’ (NCMD). The NCMD traces provided a valid way of comparing the amplitude of pupillary responses between collision and no-collision trials. It is well known that saccades trigger changes in pupillary responses, and both saccades and pupillary responses are controlled by interconnected neural circuits [1]. Therefore, in addition to characterizing average pupillary responses associated with collision and non-collision trials, we characterized the average pupillary responses triggered by saccades (saccade-locked pupil responses, [15]). Following the approach suggested by Engbert & Mergenthaler, we employed an algorithm to identify saccade times based on eye velocity [35]. The algorithm utilized multiple velocity thresholds from 1 to 40°/s (1, 5, 10, 15, 20, 25, 30, and 40°/s, with an average of 18.25°/s ± 5.37°/s) and imposed a minimal saccade duration of 2 s [1]. This algorithm extracted various saccade characteristics, including onset, duration, peak velocity, horizontal and vertical components and amplitudes [4,35]. Once identifying the onset of saccade times, we clipped the pupillary responses by creating a time window spanning 20 frames (~333 ms) before and 70 frames (~1167 ms) after the onset of each saccade. We aligned these traces and computed averages per condition for each participant. Saccade-triggered pupillary responses were standardized by either dividing or subtracting the average pupillary responses from the 10 frames (~160.5 ms) preceding the onset of each saccade. We also examined pupillary responses elicited by saccades during free viewing, establishing a baseline for our study. This reference facilitated the assessment of changes in pupil size linked to saccades, independent of users' engagement in the interception task. We employed linear interpolation during preprocessing to address gaps resulting from missing data, blink periods and outliers [1,34]. To calculate the AUC of saccade-triggered pupillary responses, we balanced between the need for accurate characterization of prolonged pupillary responses and the risk of artefact contamination from subsequent saccadic movements. Given the trade-off, we restricted the time window to 46 frames (~750 ms) following saccade detection. This approach aims to minimize artefact contamination while providing sufficient integration time for a precise estimation of the AUC.

We employed a generalized linear model (GLM) to investigate the influence of various predictors obtained in every trial on pupillary responses. Predictors included: (i) successful collision, (ii) CT, (iii) AR, (iv) *V*_T_, (v) user speed (*V*_U_, in °/s), (vi) average inter-dot distance (IDD, the distance between user and target) and (vii) the % optimal interception angle (%OIA). The IDD is crucial for analysing the participants’ performance and ability to predict and intercept the moving target [21]. The %OIA was determined by calculating the interception and tracking angular errors observed in each frame [21]. The %OIA is crucial as it offers a quantifiable measure of a participant’s capacity to anticipate and adjust to the moving target’s trajectory, a fundamental aspect of visuomotor coordination, where a higher %OIA indicates a more effective prediction of the target’s future location. In a second approach, the GLM was used to predict pupillary responses based on predictors obtained at a shorter time scale (acquired on every frame), including (i) *V*_T_, (ii) *V*_U_, (iii) [*V*_T_ *− V*_U_], (iv) *V*_G_ (gaze speed), (v) IDD, (vi) gaze-to-target distance (GTD) and (vii) %OIA. We extended our GLM analysis by incorporating two additional model fits, investigating the impact of past trials/frames on the currently observed pupillary response. The past trial influence model utilized a window of 10 preceding trials, whereas the past frame influence model employed a window of 50 previous frames.

Precise pupil-size assessment can be challenging because of the pupillary foreshortening error (PFE), which arises when the eye moves away from the camera, distorting the apparent pupil size [29,36]. To ensure that the PFE did not influence our experimental findings, we applied a correction proposed by Hayes & Petrov, known to effectively mitigate the effects of PFE [36]. To apply this correction, we estimated *A*_0_, the angular area subtended by the pupil in the baseline configuration (eye directed to the camera), as follows:


(2.1) $$A_{0}=S_{f}\cdot A\left(x,y\right),$$

where the scaling or correction factor was determined by:


(2.2) $$S_{\text{f}}=\left(\frac{1}{\sqrt{\text{cos}\theta \left(x,y\right)}}\right)$$

and the cosine of the oblique angle was given by:


(2.3) $$\text{cos}\theta \left(x,y\right)=\frac{C_{x}T_{x}+C_{y}T_{y}+C_{z}T_{z}}{\sqrt{C_{x}^{2}+C_{y}^{2}+C_{z}^{2}}\sqrt{T_{x}^{2}+T_{y}^{2}+T_{z}^{2}}},$$

where the camera lens was located at point *C*, defined by coordinates (*C_x_*, *C_y_*, *C*_z_) and gaze position on the projection screen was represented by (*T_x_*, *T_y_*, *T*_z_). Electronic supplementary material, figure S5, illustrates the experimental layout and geometric model from which we calculated *S*_f_.

### 2.5. Statistical analysis

In a pilot test with 10 participants measuring pupillary responses averaged over 20 trial repetitions and contrasting extreme AR values of 0° and 180°, we used a Student’s *t*‐test for matched pairs and calculated Cohen’s *d*, obtaining a medium effect size of 0.581. Based on this effect size, we conducted a power analysis for differences between two dependent means (matched pairs) [37], assuming an *α* = 0.05, *β* = 0.95 and an effect size of 0.581. This analysis indicated a minimum required sample size of 41 participants. We employed ANOVA tests to compare multiple samples and assess the equality of means across groups. To evaluate whether predictors explained the variability in the dependent variable, we used linear regression models combined with ANOVA tests to assess the overall significance of these models. We used a GLM (with residual sum of squares) to conduct multiple regression analyses predicting pupillary responses. This approach allowed us to assess the influence of various predictor variables on pupillary responses. Variants of the model incorporated either a history of 10 trials or 50 frames (~800 ms) to estimate the influence of past predictors on current pupillary responses [38,39]. To determine the significance of the model’s coefficients, we repeated GLM fits with random permutations of predictors 50 times to establish a reference for coefficients derived from chance [38]. This approach tests the null hypothesis (*H*_0_) that the independent variables do not affect the dependent variable, meaning the coefficients are zero. The alternative hypothesis would be that at least one independent variable does significantly predict the dependent variable. Positive coefficients indicate that past predictors increased the amplitude of pupillary responses, while negative coefficients suggest a decrease. We report our group data as mean values with standard error of the mean (s.e.m.), and statistical significance was determined at a threshold of *p *≤ 0.05. We report the test statistic (*F*-statistic along with the degrees of freedom for the model and residuals), and the *p*-value for the main effect for analysed factors. We applied Bonferroni corrections to control for type I errors following some tests. Figure panels indicate the number of participants for each analysis in parentheses.

## 3. Results

### 3.1. Trial-by-trial changes in directional uncertainty influence pupillary responses

**Figure 1.**
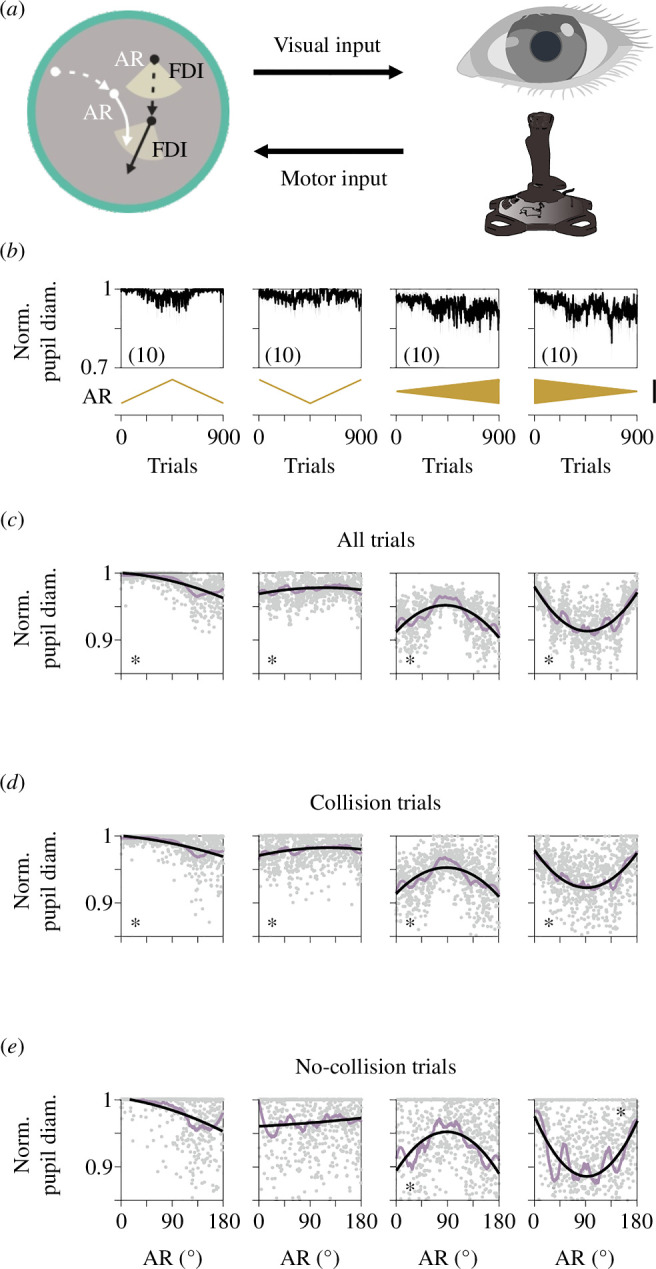
Pupillary responses to changes in directional uncertainty in the interception task. (*a*) Task overview: Participants controlled a white dot with a joystick, intercepting a black dot moving with directional changes [21]. (*b*) Average pupillary diameters across 900 trials, with lower insets showing directional uncertainty variations (ochre). Each column in the figure represents distinct uncertainty conditions characterized by consistent AR values across all panels within that column. Panels (*c*), (*d*) and (*e*) depict normalized pupillary diameters against uncertainty for all, collision and no-collision trials, respectively. Grey dots represent participant responses, with quadratic fits (black lines) and Savitzky–Golay filters (lavender lines). Asterisks denote significant fits (*p *< 0.05), details in electronic supplementary material, table S1. Number of participants is indicated in parentheses.

We employed a visuomotor task where participants chased a single moving target to investigate the impact of directional changes in the target’s motion on pupillary responses (figure 1*a*). We randomly assigned participants to one of four groups, each experiencing different AR trajectories across 900 trials. In the context of our task, the AR controls the directional uncertainty by determining the range of directions in which the target can move. A smaller AR corresponds to less directional uncertainty, as the target’s possible motion is confined to a narrower range of angles. Conversely, a larger AR increases directional uncertainty, as the target can move in a wider range of directions [21]. The panels of figure 1*b* depict the temporal progression of pupillary diameters in response to changes in AR values (lower insets), providing an overview of pupillary changes throughout the four groups. The first two groups involved small monotonic changes in AR. Initially, we employed linear models to assess the dependency and linear relationship between pupillary responses and AR. However, recognizing the possibility of curvature in the data, we also employed quadratic models to capture any potential nonlinear nature of this relationship. Pupillary diameters extracted from all trials were differentially sensitive to the AR values for participants tested with ascending–descending (linear regression, *m* = −2.07 × 10^−4^, *p* < 0.001; quadratic regression, *a* = −8.01 × 10^−7^, *p *< 0.001, *b* = −6.35 × 10^−5^, *p* = 0.04, first panel in figure 1*c*) and with descending–ascending (linear regression, *m* = 3.42 × 10^−5^, *p *< 0.001; quadratic regression, *a* = −6.98 × 10^−7^, *p* = 0.001, *b* = 1.61 × 10^−4^, *p *< 0.001, second panel in figure 1*c*) AR ramps. These results suggest that the history of AR changes influenced the evolution of the average pupillary diameter, illustrating the control exerted by AR on the observed results. The remaining two groups experienced trajectories featuring oscillating AR gradients characterized by abrupt and alternating changes in polarity with increasing or decreasing magnitudes [40]. Significant differences emerged in pupillary responses when comparing increasing (linear regression, *m* = −5.01 × 10^−5^, *p* = 0.002; quadratic regression, *a* = −5.43 × 10^−6^, *p* < 0.001, *b* = 9.27 × 10^−4^, *p *< 0.001, third panel in figure 1*c*) against decreasing (linear regression, *m* = −4.83 × 10^−5^, *p* = 0.010; quadratic regression, *a* = 7.67×10^−6^, *p *< 0.001, *b* = −1.41 × 10^−4^, *p *< 0.001, fourth panel in figure 1*c*) AR gradients. The distinct curvatures observed, an inverted quadratic function (inverted U), and a positive curvature (U-shaped) illustrate the contrasting pupillary responses to AR changes. The inverted quadratic function indicates peak responses at intermediate AR values, while the positive curvature indicates increased responses at low and high AR levels, with minimal change at intermediate AR values. These drastic differences suggest varying strategies in response to AR trajectories, highlighting adaptive processing dynamics. Furthermore, the findings reveal that manipulating the magnitude and direction of AR gradients led to higher order effects in pupillary responses, suggesting the retention of valuable information from preceding trials. The panels in figure 1*d,e* display the data and corresponding fits for pupillary responses isolated from collision (figure 1*d*) and no-collision trials (figure 1*e*). These plots offer a comparative view, illustrating the differential sensitivity across groups. In electronic supplementary material, table S1, we provide the model coefficients and outcomes of statistical tests for all trials, including collision and no-collision trials. Additionally, we illustrate the average performance and CTs of participants solving the task in electronic supplementary material, figure S1a. These plots illustrate how changes in AR influenced task performance, consistent with previous findings [21]. The observed pupillary responses to AR highlight the intricate relationship between attentional focus and the dynamics of target interception.

### 3.2. Pupillary responses to sinusoidal variations in directional uncertainty and target speed

**Figure 2.**
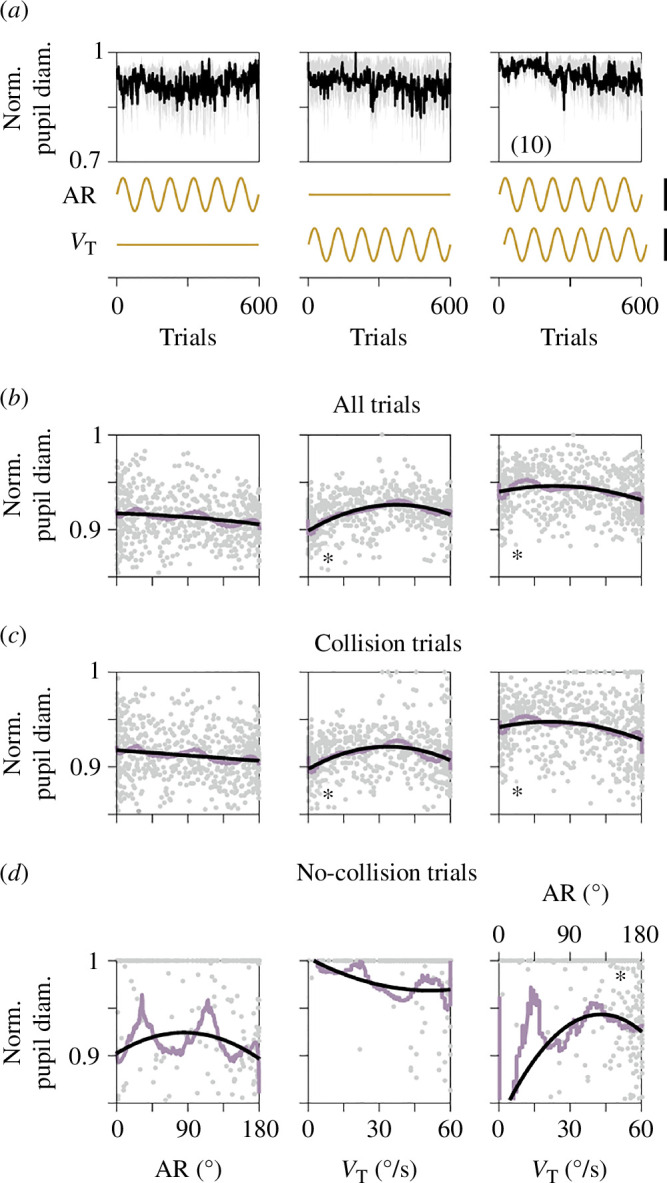
Pupillary responses to sinusoidal changes in directional uncertainty and target velocity. (*a*) Average pupillary responses across 600 trials for three experimental groups (from E2) showing changes in AR, *V*_T_ or both. Insets depict variations in AR and *V*_T_ over trials (ochre). Each column in the figure represents consistent AR and/or *V*_T_ values, which remain uniform across all panels within that column. Panels (*b*), (*c*) and (*d*) display normalized pupillary diameters against AR/*V*_T_ for all, collision and no-collision trials. Grey dots represent participant responses, with quadratic fits (black lines) and Savitzky–Golay filters (lavender lines). Asterisks indicate significant fits (*p* < 0.05), details are in electronic supplementary material, table S2. Number of participants is indicated in parentheses.

Together with AR, the target speed (*V*_T_) influences the difficulty of the interception task [21]. Therefore, distinct levels of *V*_T_ have the potential to elicit variations in perceptual processing, attentional engagement and motor planning, all of which could impact pupillary responses. We conducted experiments where AR, *V*_T_ or both were systematically varied during 600 trials using a sinusoidal function (a total of six full cycles; figure 2*a*). This sinusoidal function represents an intermediate condition between the linear ramps and the oscillating AR gradients we employed in the previous section (data from E1). Therefore, this approach was based on intermediate fluctuations in task parameters to assess their influence on average pupillary responses. By plotting pupillary responses against the independent variables (lower panels in figure 2*b–d*), we confirmed that increasing AR was associated with a reduction in average pupillary responses (linear regression, *m* = − 6.51 × 10^−5^, *p *< 0.001; quadratic regression, *a* = −2.26 × 10^−7^, *p* = 0.54, *b* = −2.42 × 10^−5^, *p* = 0.72, figure 2*b*). In contrast, *V*_T_ displayed an inverted U-shaped relationship, with maximal pupillary diameters occurring at intermediate speeds (linear regression, *m* = 3.48 × 10^−4^, *p *< 0.001; quadratic regression, *a* = −2.92 × 10^−7^, *p* < 0.001, *b* = 2.4 × 10^−5^, *p* < 0.001, figure 2*b*). This nonlinear relationship implies that *V*_T_ modulates pupillary responses distinctly across various difficulty levels. Intermediate *V*_T_ may enhance alertness and cognitive engagement, maximizing pupillary dilation. Notably, when both *V*_T_ and AR were varied simultaneously, the effects on pupillary responses became less pronounced (AR: linear regression, *m* = −5.11 × 10^−5^, *p *< 0.001; quadratic regression, *a* = −1.25 × 10^−6^, *p *< 0.001, *b* = 1.75 × 10^−5^, *p *< 0.005; *V*_T_: linear regression, *m* = 3.21 × 10^−5^, *p* = 0.56; quadratic regression, *a* = 9.97 × 10^−7^, *p* = 0.82, *b* = −3.76 × 10^−5^, *p* = 0.91, figure 2*b*). This diminished impact was unexpected and contrasted with the pronounced effects observed with the oscillating AR gradients alone. It suggests a nonlinear interaction between these two variables. Further experimentation involving slower frequencies and varying phases between sinusoidal functions will be required to explore this possibility. The model coefficients and statistical tests for all, collision and no-collision trials depicted in figure 2*b–d* can be referenced in electronic supplementary material, table S2. Average performance and CTs are illustrated in electronic supplementary material, figure S1b. Therefore, the sinusoidal variations in AR and *V*_T_ distinctly influenced pupillary dynamics.

### 3.3. Behavioural history influences pupillary responses

**Figure 3.**
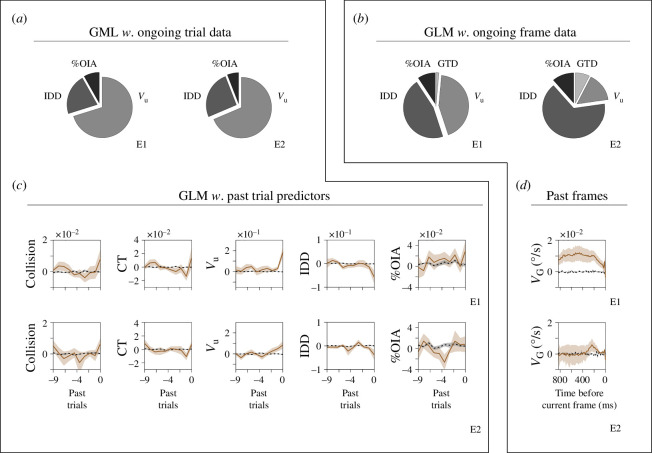
Influence of current and past behavioural metrics on pupillary responses. (*a*) Pie charts illustrate the relative weights of regression coefficients computed using average values from ongoing trial data. Data analysis from experiments where only AR varied across trials (*e1*) revealed that *V*_U_, and %OIA were significant predictors of pupillary responses. Similarly, in experiments where AR, *V*_T_ or both varied (*e2*) *V*_T_ and IDD were significant predictors. (*b*) Analysis like (*a*) but using 'fast predictors', namely averages of behavioural data obtained on the currently acquired frame. (*c*) Line plots illustrate the weighted coefficients from a generalized linear model (GLM) associated with different predictors across ten past trials leading up to the current trial. These results support the notion that pupillary responses depend on these past predictors. (*d*) Same as (*c*) but using a history of past 50 frames, and their statistical significance was determined using a permutation test that shuffled the trial/frame order 50 times for the variable of interest (black lines) to yield a *p*-value for the permutation test.

The relationships observed between pupillary responses and AR when participants were exposed to increasing and decreasing AR gradients suggest higher order effects that might retain information from preceding trials. The well-established choice history bias, documented in the literature for over a century, posits that decisions are influenced by sensory evidence and the history of preceding choices [39,41–43]. We hypothesized that analogous historical influences might also manifest in the gradual changes observed in pupillary responses during our experiments. We employed GLM to explore how current and past behavioural metrics influenced pupillary responses [38,39]. GLM considers current and past predictors, allowing for capturing intricate patterns of pupillary responses on a response-by-response basis [38,44]. In a first approach, we calculated regression coefficients using only the ongoing trial’s data. Analysis from experiments where only AR varied across trials (i.e. data from E1) revealed that *V*_U_ and %OIA were significant predictors of pupillary responses (*V*_U_, ANOVA, *F*_1,17_ = 14.28, *p* < 0.001; IDD, *F*_1,17_ = 2.52, *p* = 0.11; %OIA, *F*_1,17_ = 5.14, *p* = 0.02, figure 3*a*). Similarly, in experiments where AR, *V*_T_ or both varied (i.e. data from E2), *V*_U_ and IDD were significant predictors (*V*_U_, *F*_1,17_ = 13.16, *p* < 0.001; IDD, *F*_1,17_ = 3.86, *p* = 0.04; %OIA, *F*_1,17_ = 0.21, *p* = 0.65, figure 3*a*). In a second approach, we predicted the pupillary responses using ‘instantaneous’ data gathered from the current frame within the ongoing trial. Results indicated that *V*_U_, IDD and %OIA were strong predictors of pupillary responses in experiments with AR variation (E1: *V*_U_, *F*_1,17_ = 10.82, *p* < 0.001; IDD, *F*_1,17_ = 14.28, *p* < 0.001; GTD, *F*_1,17_ = 0.57, *p* = 0.44; %OIA, *F*_1,17_ = 14.28, *p* = 0.02, left pie chart in figure 3*b*), while *V*_U_, IDD, GTD and %OIA predicted pupillary responses in experiments where AR, *V*_T_ or both varied (E2: *V*_U_, *F*_1,17_ = 7.20, *p* < 0.007; IDD, *F*_1,17_ = 14.28, *p* < 0.001; GTD, *F*_1,17_ = 5.49, *p* = 0.01; %OIA, *F*_1,17_ = 7.82, *p* = 0.005, right pie chart in figure 3*b*).

Integrating current and past predictors in GLM analysis revealed that successful collision, collision time, *V*_U_ and %OIA predicted pupillary responses in experiments with AR changes (E1: successful collision, ANOVA, *F*_1,17_ = 4.48, *p* = 0.03; reach time, *F*_1,17_ = 5.14, *p* = 0.02; *V*_U_, *F*_1,17_ = 14.28, *p *< 0.001; IDD, *F*_1,17_ = 2.28, *p* = 0.13; %OIA, *F*_1,17_ = 4.16, *p* = 0.04, upper panels in figure 3*c*). However, in experiments with AR, *V*_T_, or both variations, only *V*_U_ predicted pupillary responses (E2: successful collision, *F*_1,17_ = 2.28, *p* = 0.13; reach time, *F*_1,17_ = 0.28, *p* = 0.59; *V*_U_, *F*_1,17_ = 11.57, *p *< 0.001; IDD, *F*_1,17_ = 3.02, *p* = 0.08; %OIA, *F*_1,17_ = 1.46, *p* = 0.22, lower panels in figure 3*c*). When using the ‘fast predictors,’ *V_G_* and IDD predicted pupillary responses in experiments where AR was varied across trials (E1: *V*_G_, *F*_1,17_ = 4.81, *p* = 0.03; IDD, *F*_1,17_ = 7.60, *p* = 0.008, upper panel in figure 3*d*). In experiments where AR, *V*_T_ or both varied, *V*_G_ and GTD predicted pupillary responses (E2: *V*_G_, *F*_1,17_ = 6.60, *p* = 0.01; IDD, *F*_1,17_ = 0.14, *p* = 0.71; GTD, *F*_1,17_ = 5.49, *p* = 0.01, lower panel in figure 3*d*). These results demonstrate that participant-controlled measures had a significant historical influence on pupillary responses. *V*_U_ and IDD had a critical impact on pupillary responses, with %OIA and GTD also playing a role, suggesting a predictive component in the task [21,45]. The analysis presented in this section demonstrates that behavioural parameters from the current and immediately preceding trials strongly predict pupillary responses in the current trial. Additionally, with slowly varying inter-trial changes in AR, *V*_G_ showed a robust predictive relationship with pupillary responses. However, this predictive power was attenuated in experiments involving faster intra-trial variations in AR. These results illustrate the importance of preceding behavioural context in shaping pupillary responses.

### 3.4. Pupillary responses decrease with directional uncertainty

**Figure 4.**
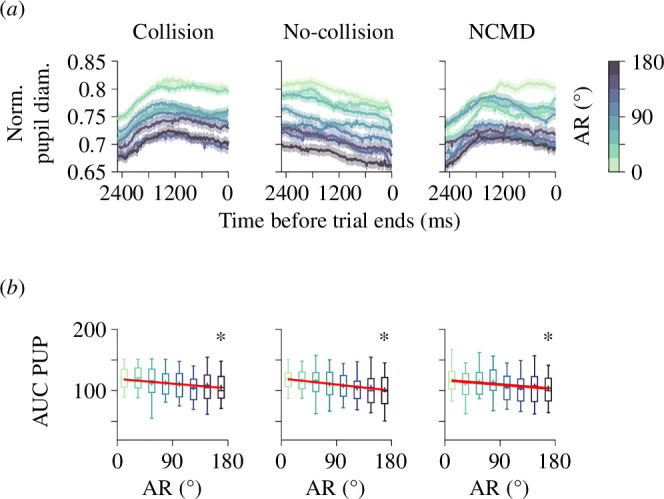
Directional uncertainty reduces pupillary responses in our interception task. (*a*) Normalized pupillary responses, aligned to the end of interception trials, are illustrated for collision (left), no-collision (middle) trials and NCMD (right). The data for this analysis corresponds to the two first groups of E1, assessed with ascending–descending and descending–ascending AR ramps. (*b*) Lower panels show boxplots of the AUC of pupillary responses as a function of AR for collision (left), no-collision (middle) and NCMD (right) trials. The asterisks indicate significant slopes in the linear regression models. Complementary panels and the same analysis performed on the two remaining groups from E1 (i.e. those with increasing and decreasing AR gradients) can be found in electronic supplementary material, figure S3.

Previous research has suggested that uncertainty processing may heighten cognitive effort, leading to larger pupillary diameters [24,25,27,28]. We examined how AR and collision outcome impacted participants' pupillary responses in our target interception task, as depicted in the averaged traces aligned either to the moment of collision or to the end of the trial in cases with no collision. Our findings revealed a prototypic dependence of pupillary responses on directional uncertainty as participants approached the end of the trial (analysis performed on the first two groups of E1 in figure 4*a*). We utilized the AUC of pupillary traces to compare the overall effects of directional uncertainty on pupillary responses. Higher uncertainty values were associated with a decrease in AUC of pupillary responses, regardless of whether the trial ended in a collision or no collision (AUC PUP versus AR: linear regression model w. ANOVA test: collision: *m* = −8.51 × 10^−2^, *F*_1,6_ = 32.80, *p* < 0.001; no-collision: −10.97 × 10^−2^, *F*_1,6_ = 210.79, *p* < 0.001; figure 4*b*). Therefore, these findings show that, in our task, pupillary responses decreased with AR, irrespective of the collision outcome. A potential issue when comparing collision and no-collision trials is the inherent difference in trial durations, which may impact the validity of the analysis. Indeed, in our case, no-collision trials always lasted 5 s (i.e. maximal trial duration), resulting in absolute trial duration differences. Thus, our concern was that the trial duration difference could potentially explain the reduction in pupillary diameters with AR. We implemented an analytical approach that clipped the pupillary response traces from no-collision trials to match the duration of the collision trials. We refer to these traces as ‘NCMD’ (figure 4*a*). The NCMD pupillary traces confirmed the negative relationship between pupillary responses and directional uncertainty (linear regression model w. ANOVA test: collision: *m* = −8.51 × 10^−2^, *F*_1,6_ = 23.06, *p* < 0.001; figure 4*b*). This analysis suggests that the reduction in pupillary responses with higher directional uncertainty in error trials was not an artefact of trial duration differences. Matching the durations of collision and no-collision trials (through NCMD trials) enabled valid comparisons across groups and facilitated assessing the impact of independent variables on pupillary responses. Electronic supplementary material, figure S3, offers further details on the detection and comparison of NCMD traces, along with a similar analysis applied to the last two experimental groups from E1. The decrease in pupillary responses with AR in our target-chasing task provides a new clue on how the brain adapts to unpredictable conditions.

### 3.5. Pupillary diameter estimation with free viewing in our interception task

In pupillometry research, fixed gaze and central fixations are standard practices because they tend to produce optimal eye-tracking accuracy. Central fixations provide consistent luminance and contrast levels, reducing potential confounding effects on pupillary diameter [46]. Moreover, maintaining a fixed gaze aids in reducing artefacts and minimizing PFE, which is a distortion occurring when the eyes rotate away from the camera [36]. In our experiments, one possibility could be that a change in the frequency of saccadic movements with directional uncertainty (AR) could explain the observed reduction in pupillary diameters. Moreover, participant-controlled viewing might have increased the misestimation of pupillary responses, especially at the edges or periphery of the arena. If so, this could result in a systematic reduction in estimated pupillary diameters, potentially explaining the observed decrease in pupillary responses linked to the directional uncertainty of the target (figure 4*b*).

**Figure 5.**
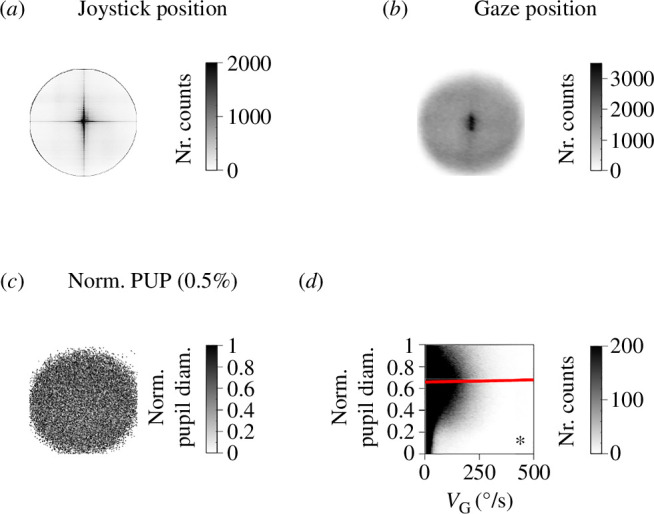
Pupillary responses: centre dominance and radial independence in task gaze positions. Combined data from E1 and E2. (*a*) Two-dimensional density map of joystick positions, concentrated at the centre. (*b*) Density map for gaze positions, showing focus on the central screen. (*c*) Fraction (0.5%, randomly chosen) of pupillary diameters across arena locations, indicating no consistent trend relative to the radius. (*d*) Normalized pupillary diameter versus gaze speed (*v*_G_), with a positive slope (red regression line). Density plots with 200 bins per axis.

We calculated the two-dimensional frequency distributions of joystick and gaze positions (pooled data from E1 and E2). Both plots revealed a clear peak at the arena’s centre, indicating that joystick and gaze responses tended to occur at the centre of the screen (figure 5*a,b*). These findings demonstrate that our task effectively balanced stimulus positions. Therefore, while the stimulus appeared at various locations throughout the task, these presentations were consistently counterbalanced across trials. Moreover, by plotting a randomly selected subset of all pupillary diameter versus gaze position pairs (0.5% of the entire database, randomly chosen), we found that there was no systematic bias in the estimation of pupillary diameter along the radius of the arena (figure 5*c*), suggesting minimal or no influence of PFE in our task [36]. Therefore, these findings showed minimal bias in pupil diameters across radial positions within the arena. Moreover, while the camera was situated at the bottom rather than the centre of the monitor, which could introduce the possibility of biases towards the top of the arena, our findings suggest no systematic effects in that direction. Finally, plotting pupillary responses as a function of instantaneous gaze speed showed a net positive slope (linear regression w. ANOVA test, *m* = 3.78 × 10^−5^, *F* = 384.27, *p *< 0.001; figure 5*d*). This finding indicates that increased average gaze speed (as would occur with more saccades) did not reduce the estimated pupillary diameter. These results suggest that, in our experimental conditions, saccades or the PFE were unlikely to lead to strong misestimations of pupillary diameter. The central dominance and radial independence in task gaze positions support our experimental setup, ensuring minimal bias and validating the reliability of our pupillary diameter estimations.

### 3.6. Saccade-triggered pupillary responses and directional uncertainty

Our findings reveal a negative relationship between average pupillary responses and directional uncertainty. However, multiple saccades occurred during interception trials, and one possibility is that pupillary responses triggered by saccades may respond differently to increasing AR values. Saccades, involving higher cognitive processes like attention and decision-making, may provide specific insights into how the eye and brain respond to directional uncertainty. Thus, while average pupillary responses offer a broad measure of pupil reaction, saccade-triggered pupillary responses could reveal unique aspects of this response [6,11,15,47,48].

**Figure 6.**
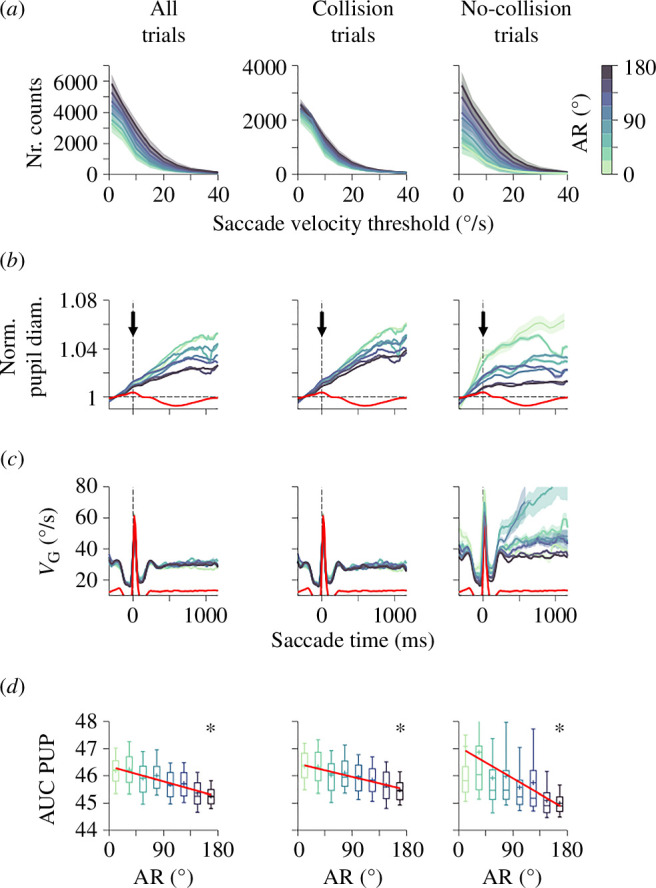
Effect of directional uncertainty on saccade-triggered pupillary responses in our interception task. This figure presents the analysis of saccade-triggered pupillary responses from Experiment 1 (E1). Organized in columns for all, collision and no-collision trials, the panels depict the number of detected cases as a function of saccade velocity threshold (*a*), normalized saccade-triggered pupillary diameters (*b*), gaze speed (*V*_G_) (*c*) and AUC under the pupillary responses as a function of AR (*d*). Different AR values are represented in colours, with a corresponding colour bar on the right. Red pupillary traces correspond to average responses obtained during the fourth calibration routine (i.e. saccades during free viewing), establishing a baseline reference for saccade-triggered pupillary changes without active target tracking. The asterisks indicate significant slopes in the linear regression models.

We utilized an algorithm relying on eye velocity measurements [35] to identify saccade events across various experimental conditions. This algorithm was based on multiple relative velocity thresholds ranging from 1 to 40°/s and imposed a minimal saccade duration of 2 s (see §2). Intuitively, as tasks become more challenging, one might anticipate a reduction in saccade production due to the increased concentration of gaze to manage task difficulty. However, in our task involving a single moving target, it is possible that directional uncertainty could prompt participants to escalate their eye movements and saccades to intercept the target. Panels from figure 6*a* depict the number of detected saccades as a function of the saccade velocity threshold, with colours representing distinct AR values. Indeed, as expected, higher AR values led to a greater number of detected saccades across all (left), collision (middle) and no-collision (right) trials (figure 6*a* and electronic supplementary material, figure S4). After saccade detection, we measured various saccade characteristics, including onset time, duration, peak velocity and horizontal and vertical amplitude components [4]. We extracted the saccade-triggered pupillary responses by defining a temporal window spanning 20 frames (~330 ms) before and 70 frames (~1150 ms) after each saccade event using the saccade onsets. We aligned these responses, averaged them across participants and conditions and colour-coded them according to AR (figure 6*b*). The red traces represent the averaged saccade-triggered responses obtained during the fourth calibration routine, during which we collected pupillary data while participants engaged in free eye viewing/saccade production. This procedure helped us establish a baseline reference for saccade-triggered pupillary changes without any moving target. Panels from figure 6*c* illustrate the prototypical shape of saccade-triggered gaze speeds found from the analysis. We present the onsets and durations of these saccades relative to AR in electronic supplementary material, figure S4.

We calculated the AUC of normalized pupillary responses within 46 frames (a ~750 ms window) after saccade onset, capturing the cumulative pupillary response during this period. Across all experimental groups from E1, there was a consistent negative linear relationship between saccade-triggered pupillary responses and AR, indicating that pupil diameter was negatively correlated to AR (all trials; ascending–descending AR ramp, linear regression w. ANOVA test, *m* = −5.19 × 10^−3^, *F*_1,6_ = 10.55, *p* = 0.02; descending–ascending AR ramp, linear regression, *m* = −4.41 × 10^−3^, *F*_1,6_ = 13.32, *p* = 0.04; increasing AR gradients, linear regression, *m* = −1.01 × 10^−3^, *F*_1,6_ = 14.22, *p* = 0.04; decreasing AR gradients, linear regression, *m* = −12.59 × 10^−3^, *F*_1,6_ = 54.45, *p* < 0.002; not illustrated). The consistent negative slopes found across all groups prompted us to pool the data for an integrated analysis, further confirming the robust negative relationship (all trials, linear regression w. ANOVA test, *m* = −7.78 × 10^−3^, *F*_1,6_ = 67.78, *p *< 0.0005, collision trials, *m* = −6.10 × 10^−3^, *F*_1,6_ = 101.20, *p *< 0.0004, no-collision trials, *m* = −9.63 × 10^−3^, *F*_1,6_ = 53.76, *p* = 0.01; figure 6*d*). Our results indicate that saccade-triggered pupillary responses offer a unique perspective on the dynamic interaction between eye movements and cognitive processes in the brain. The negative relationship between pupillary diameter and AR suggests that heightened uncertainty imposed greater demands on the oculomotor system. This increased the frequency of saccades with smaller pupillary diameters, probably facilitating focused foveal vision.

As mentioned, accurately measuring pupil size in tasks without fixed gaze poses challenges due to the PFE. This error arises when the eye deviates from the camera’s view, leading to distortions in the recorded pupil size [29,36]. Although unlikely given our results, it remains a possibility that this distortion could explain the decreased pupillary diameter reduction observed in previous sections. Therefore, we conducted an additional analysis by integrating a corrective equation proposed by Hayes & Petrov into our study [36] (electronic supplementary material, figure S5). This equation addresses PFE by estimating how the pupil area is foreshortened based on the angle between the eye-to-camera and eye-to-stimulus axes. Incorporating this model significantly reduces the root mean squared error of pupil measurements, thereby enhancing the precision of our pupillometry assessments. Therefore, following correcting pupillary metrics for PFE across all participants, we revisited the analysis of pupillary responses aligned with the moment of collision (or the end of the trial for no-collision trials, electronic supplementary material, figure S6a). Our findings reaffirmed that increased AR resulted in a reduction in the AUC of pupillary responses, irrespective of whether the trial concluded with a collision or no collision (AUC PUP versus AR: linear regression model w. ANOVA test: collision: *m* = −9.17 × 10^−2^, *F*_1,6_ = 34.04, *p *< 0.001; no-collision: −11.38 × 10^−2^, *F*_1,6_ = 185.78, *p *< 0.001; NCMD: −6.57 × 10^−2^, *F*_1,6_ = 11.87, *p* = 0.01; electronic supplementary material, figure S6b).

Likewise, we repeated the analysis of saccade-triggered pupillary responses following PFE correction (electronic supplementary material, figure S6c), confirming a negative linear correlation between saccade-triggered pupillary responses and AR (all trials, linear regression w. ANOVA test, *m* = −2.78 × 10^−3^, *F*_1,6_ = 34.15, *p *< 0.002, collision trials, *m* = −2.18 × 10^−3^, *F*_1,6_ = 15.30, *p *< 0.007, no-collision trials, *m* = −6.89 × 10^−3^, *F*_1,6_ = 16.64, *p *< 0.006; electronic supplementary material, figure S6d). These results indicate a negative correlation between AR and pupillary diameters in our task. Lastly, we repeated our main analyses on pupillary responses using a subtractive standardization preprocessing procedure [34] instead of a divisive one, both with and without PFE correction. In all cases, we confirmed a negative relationship between pupillary responses and AR (electronic supplementary material, figure S7).

## 4. Discussion

We investigated the impact of directional uncertainty on pupillary responses in a visually guided interception task with a single moving target. Manipulating the AR controlled the uncertainty, revealing distinct influences on pupillary diameters. Quadratic curves for increasing and decreasing AR gradients revealed historical influences on pupillary responses. Recent studies manipulating uncertainty levels have implicated pupil-linked brain arousal in updating mental models following unexpected changes. Indeed, changes in pupillary diameter observed along the different AR gradients might be explained by Bayesian adjustments [26]. By employing such a probabilistic approach, participants can estimate the likelihood of various outcomes, accommodating uncertainty and changes over time. Thus, in the context of our study, it suggests that the brain utilizes past information to update its predictions about future events, which is reflected in the evolution of pupillary responses. Furthermore, pupil diameter reflects global gain modulation, influencing decision strategies mediated by neuromodulator systems [12,49]. Our exploration extended to sinusoidal variations in AR, *V*_T_ or both, showing robust effects on pupillary responses. However, the combined impact of AR and *V*_T_ appeared less pronounced, suggesting nonlinear interactions. Investigating lower frequency variations and potential nonlinear interactions presents future research opportunities, highlighting pupillary responses as real-time indicators in interception tasks. Sensory prediction errors play a crucial role in motor adaptation, particularly in response to variations in *V*_T_ [50]. Our task generates two outcomes from voluntary motor commands: sensory consequences observed through vision and proprioception and subjective assessments of trial execution. Our findings indicate that sensory prediction errors drive implicit recalibration, influencing adjustments in task errors and facilitating explicit learning [51,52]. Future experiments should further quantify this interaction to confirm its significance and validity. Recent behavioural history significantly influenced pupillary responses, suggesting a cumulative record of experiences and actions [38,39,44]. Our use of GLM fits involved testing the potential influence of multiple variables on pupillary responses, allowing us to empirically identify predictors. In future studies, we could refine our model by systematically selecting variables based on established theoretical frameworks. Pupillary responses decreased with AR, confirming their negative relationship. Saccade-triggered pupillary responses further demonstrated this negative correlation, providing insights into uncertainty processing during eye movements. Therefore, our findings revealed reduced pupillary responses with directional uncertainty in a foveal task based on intercepting a moving target.

Our study contrasts with findings from Donner’s lab, where increased pupillary diameters were found in response to uncertainty, an observation consistent with adaptive gain theory [23,27,53]. According to this theory, choices involving potential future rewards are associated with larger baseline pupil diameters than choices focused on known high payoffs. In contrast, our study shows a decrease in pupillary responses with AR, alongside larger saccade-triggered pupillary responses in collision trials compared with no-collision trials. Variations in response time distributions between correct and incorrect responses could explain the differences in pupillary diameters, highlighting potential confounding factors across experimental setups [54–56]. Methodological considerations, including task design, could further influence these outcomes, as pupil responses are intricately shaped by the specific demands of each perceptual task [4,27,57–59]. Although our analysis demonstrated that average gaze speed did not decrease the estimated pupillary diameter, and our results remained consistent across different preprocessing methods of pupillary signals, including PFE correction, we acknowledge the possibility that the increased number of saccades with higher AR may be related to the observed reduction in pupillary diameter.

In our study, the requirement of detailed central vision favours small pupils, which enhances acuity. Conversely, large pupils optimize peripheral vision by increasing light intake and sensitivity, particularly advantageous for tasks like the coherence dot motion task [27,55]. Numerous studies have explored the relationship between pupillary diameter and attention [60], revealing its sensitivity to lapses in attention [61,62]. However, this relationship is complex, as smaller pupils can enhance visual acuity and improve attentional performance [62]. For example, reduced pupil size has been linked to better discrimination of fine details, which aids in tasks requiring focussed attention [57]. The dynamic nature of pupil size changes, involving both constriction and dilation, suggests that visual perception is finely tuned to task demands, with adjustments in pupil size facilitating optimal performance rather than maintaining a constant state of constriction [57]. Therefore, pupil size reflects a delicate balance between visual sensitivity (which benefits from larger pupils in low-light conditions) and visual acuity (which is supported by smaller pupils in bright conditions) [18]. However, distinguishing between peripheral and foveal vision remains challenging due to their interconnected nature [63].

In the field of decision-making, researchers have explored various forms of uncertainty [22]. Expected uncertainty arises when there is limited information about predicted outcomes, while unexpected uncertainty results from unforeseen changes in learned contingencies. Across these studies, uncertainty processing is intricately tied to task performance [22]. Our task was no exception in this regard, as higher levels of uncertainty are associated with lower performance [21]. In our experimental setup, task difficulty was manipulated through parameters such as *v_T_* and FDI, which determined the speed and frequency of target movement direction changes. AR reflects the unpredictability of these changes, introducing variability and ambiguity that increase the perceptual-motor challenge. These factors (*V*_T_, FDI, AR) collectively influence participants' ability to intercept the moving target [21]. Therefore, we believe that disentangling uncertainty processing from task performance is unfeasible in our study due to their close interconnection. This conflation of task difficulty and AR complicates our ability to attribute variations in pupillary responses solely to AR manipulations or task difficulty. The processing of uncertainty appears to be a fundamental aspect of cognitive control that directly influences decision-making and task execution.

Several constraints in our study merit consideration due to potential data interpretation confounds. The precision limitations of eye trackers not explicitly designed for pupillometry may affect measurement accuracy. Despite controlling for luminance conditions to address the pupillary light reflex, another significant confound is PFE, occurring as the eye rotates away from the camera, leading to elliptical pupillary images. To mitigate PFE, constant fixation could be enforced, but the nature of our task precluded this. However, stimulus–position counterbalancing was applied to minimize bias. Additionally, we employed a geometric model to estimate and correct for PFE [36], validating our finding of decreased pupillary responses with directional uncertainty. While we assumed constant physical effort during trials, variations in joystick movement effort may have influenced pupil size [25,64]. Other uncontrolled factors, such as vascular changes in the iris and rhythmic oscillations, could also affect pupil size [2,4,5,46,65]. Furthermore, we did not explore in depth the interaction between AR and *V*_T_, limiting our understanding of their combined effects on pupillary responses [21] and suggesting avenues for future research.

## 5. Conclusion

In this study, we manipulated directional uncertainty in a visuomotor task involving single-dot interception by varying AR and *V*_T_. Our findings consistently showed that higher levels of directional uncertainty, characterized by larger AR values, resulted in smaller pupillary dilation. Saccade-triggered pupillary responses followed a similar pattern, indicating reduced pupillary changes during saccades under conditions of higher uncertainty. Therefore, these results reveal a negative relationship between pupillary responses and directional uncertainty in our dynamic visuomotor task. Our results highlight the potential of pupillary responses as biomarkers reflecting cognitive processes such as attention and decision-making. Pupillary responses could inform the development of adaptive interfaces that adjust task difficulty in real-time, enhancing user experience in educational software, gaming and high-stakes environments like air traffic control while also guiding the design of tailored training programs in fields requiring precise motor coordination and rapid decision-making, such as aviation, sports and military operations.

## Data Availability

Data can be found at [66]. Supplementary material is available online [67].

## References

[1] Liversedge SP, Gilchrist I. 2011 The oxford handbook of eye movements. (ed. S Everling). UK: Oxford University Press. (10.1093/oxfordhb/9780199539789.001.0001)

[2] Steinhauer SR, Bradley MM, Siegle GJ, Roecklein KA, Dix A. 2022 Publication guidelines and recommendations for pupillary measurement in psychophysiological studies. Psychophysiology **59**, e14035. (10.1111/psyp.14035) 35318693 PMC9272460

[3] Watson AB, Yellott JI. 2012 A unified formula for light-adapted pupil size. J. Vis. **12**, 12. (10.1167/12.10.12) 23012448

[4] Mahanama B, Jayawardana Y, Rengarajan S, Jayawardena G, Chukoskie L, Snider J, Jayarathna S. 2022 Eye movement and pupil measures: a review. Front. Comput. Sci. **3**, 733531. (10.3389/fcomp.2021.733531)

[5] Beatty J, Lucero-Wagoner B. 2000 The pupillary system. In Handbook of psychophysiology, pp. 142–162, 2nd ed. New York: Cambridge University Press.

[6] Burlingham CS, Mirbagheri S, Heeger DJ. 2022 A unified model of the task-evoked pupil response. Sci. Adv. **8**, eabi9979. (10.1126/sciadv.abi9979) 35442730 PMC9020670

[7] Laeng B, Sulutvedt U. 2014 The eye pupil adjusts to imaginary light. Psychol. Sci. **25**, 188–197. (10.1177/0956797613503556) 24285432

[8] Unsworth N, Robison MK, Miller AL. 2018 Pupillary correlates of fluctuations in sustained attention. J. Cogn. Neurosci. **30**, 1241–1253. (10.1162/jocn_a_01251) 29488845

[9] Denison RN, Parker JA, Carrasco M. 2020 Modeling pupil responses to rapid sequential events. Behav. Res. Methods **52**, 1991–2007. (10.3758/s13428-020-01368-6) 32144729 PMC7688292

[10] Geva R, Zivan M, Warsha A, Olchik D. 2013 Alerting, orienting or executive attention networks: differential patters of pupil dilations. Front. Behav. Neurosci. **7**, 145. (10.3389/fnbeh.2013.00145) 24133422 PMC3796264

[11] de Gee JW, Knapen T, Donner TH. 2014 Decision-related pupil dilation reflects upcoming choice and individual bias. Proc. Natl Acad. Sci. USA **111**, E618–25. (10.1073/pnas.1317557111) 24449874 PMC3918830

[12] Murphy PR, Boonstra E, Nieuwenhuis S. 2016 Global gain modulation generates time-dependent urgency during perceptual choice in humans. Nat. Commun. **7**, 13526. (10.1038/ncomms13526) 27882927 PMC5123079

[13] de Gee JW, Colizoli O, Kloosterman NA, Knapen T, Nieuwenhuis S, Donner TH. 2017 Dynamic modulation of decision biases by brainstem arousal systems. eLife **6**, e23232. (10.7554/eLife.23232) 28383284 PMC5409827

[14] Van Slooten JC, Jahfari S, Knapen T, Theeuwes J. 2018 How pupil responses track value-based decision-making during and after reinforcement learning. PLoS Comput. Biol. **14**, e1006632. (10.1371/journal.pcbi.1006632) 30500813 PMC6291167

[15] Knapen T, de Gee JW, Brascamp J, Nuiten S, Hoppenbrouwers S, Theeuwes J. 2016 Cognitive and ocular factors jointly determine pupil responses under equiluminance. PLoS One **11**, e0155574. (10.1371/journal.pone.0155574) 27191166 PMC4871560

[16] Zuber BL, Stark L, Lorber M. 1966 Saccadic suppression of the pupillary light reflex. Exp. Neurol. **14**, 351–370. (10.1016/0014-4886(66)90120-8) 4951848

[17] Stewart EEM, Valsecchi M, Schütz AC. 2020 A review of interactions between peripheral and foveal vision. J. Vis. **20**, 2. (10.1167/jov.20.12.2) PMC7645222 33141171

[18] Vilotijević A, Mathôt S. 2024 Functional benefits of cognitively driven pupil-size changes. Wiley Interdiscip. Rev. Cogn. Sci. **15**, e1672. (10.1002/wcs.1672) 38149763

[19] Hafed ZM, Goffart L, Krauzlis RJ. 2009 A neural mechanism for microsaccade generation in the primate superior colliculus. Science **323**, 940–943. (10.1126/science.1166112) 19213919 PMC2655118

[20] Wang CA, Blohm G, Huang J, Boehnke SE, Munoz DP. 2017 Multisensory integration in orienting behavior: pupil size, microsaccades, and saccades. Biol. Psychol. **129**, 36–44. (10.1016/j.biopsycho.2017.07.024) 28789960

[21] Treviño M, Medina-Coss y León R, Támez S, Beltrán-Navarro B, Verdugo J. 2024 Directional uncertainty in chase and escape dynamics. J. Exp. Psychol. **153**, 418–434. (10.1037/xge0001510) 37956078

[22] Bland AR, Schaefer A. 2012 Different varieties of uncertainty in human decision-making. Front. Neurosci. **6**, 85. (10.3389/fnins.2012.00085) 22701401 PMC3370661

[23] Jepma M, Nieuwenhuis S. 2011 Pupil diameter predicts changes in the exploration-exploitation trade-off: evidence for the adaptive gain theory. J. Cogn. Neurosci. **23**, 1587–1596. (10.1162/jocn.2010.21548) 20666595

[24] Lavín C, San Martín R, Rosales Jubal E. 2013 Pupil dilation signals uncertainty and surprise in a learning gambling task. Front. Behav. Neurosci. **7**, 218. (10.3389/fnbeh.2013.00218) 24427126 PMC3879532

[25] Zénon A. 2019 Eye pupil signals information gain. Proc. Biol. Sci. **286**, 20191593. (10.1098/rspb.2019.1593) 31530143 PMC6784722

[26] Treviño M, Castiello S, De la Torre‐Valdovinos B, Osuna Carrasco P, Medina‐Coss y León R, Arias‐Carrión O. 2023 Two‐stage reinforcement learning task predicts psychological traits. Psych J. **12**, 355–367. (10.1002/pchj.633) 36740455

[27] Urai AE, Braun A, Donner TH. 2017 Pupil-linked arousal is driven by decision uncertainty and alters serial choice bias. Nat. Commun. **8**, 14637. (10.1038/ncomms14637) 28256514 PMC5337963

[28] Vincent P, Parr T, Benrimoh D, Friston KJ. 2019 With an eye on uncertainty: modelling pupillary responses to environmental volatility. PLoS Comput. Biol. **15**, e1007126. (10.1371/journal.pcbi.1007126) 31276488 PMC6636765

[29] Krejtz K, Duchowski AT, Niedzielska A, Biele C, Krejtz I. 2018 Eye tracking cognitive load using pupil diameter and microsaccades with fixed gaze. PLoS One **13**, e0203629. (10.1371/journal.pone.0203629) 30216385 PMC6138399

[30] Richer F, Beatty J. 1987 Contrasting effects of response uncertainty on the task-evoked pupillary response and reaction time. Psychophysiology **24**, 258–262. (10.1111/j.1469-8986.1987.tb00291.x) 3602280

[31] Treviño M, Castiello S, Arias-Carrión O, De la Torre-Valdovinos B, Coss Y León RM. 2021 Isomorphic decisional biases across perceptual tasks. PLoS One **16**, e0245890. (10.1371/journal.pone.0245890) 33481948 PMC7822501

[32] Treviño M. 2020 Non-stationary salience processing during perceptual training in humans. Neuroscience **443**, 59–70. (10.1016/j.neuroscience.2020.07.011) 32659341

[33] Porter G, Troscianko T, Gilchrist ID. 2007 Effort during visual search and counting: insights from pupillometry. Q. J. Exp. Psychol. **60**, 211–229. (10.1080/17470210600673818) 17455055

[34] Mathôt S, Vilotijević A. 2023 Methods in cognitive pupillometry: design, preprocessing, and statistical analysis. Behav. Res. Methods **55**, 3055–3077. (10.3758/s13428-022-01957-7) 36028608 PMC10556184

[35] Engbert R, Mergenthaler K. 2006 Microsaccades are triggered by low retinal image slip. Proc. Natl Acad. Sci. USA **103**, 7192–7197. (10.1073/pnas.0509557103) 16632611 PMC1459039

[36] Hayes TR, Petrov AA. 2016 Mapping and correcting the influence of gaze position on pupil size measurements. Behav. Res. Methods **48**, 510–527. (10.3758/s13428-015-0588-x) 25953668 PMC4637269

[37] Faul F, Erdfelder E, Lang AG, Buchner A. 2007 G*Power 3: a flexible statistical power analysis program for the social, behavioral, and biomedical sciences. Behav. Res. Methods **39**, 175–191. (10.3758/bf03193146) 17695343

[38] Treviño M. 2014 Stimulus similarity determines the prevalence of behavioral laterality in a visual discrimination task for mice. Sci. Rep. **4**, 7569. (10.1038/srep07569) 25524257 PMC5378985

[39] Treviño M, Medina-Coss y León R, Haro B. 2020 Adaptive choice biases in mice and humans. Front. Behav. Neurosci. **14**. (10.3389/fnbeh.2020.00099) PMC7372118 32760255

[40] Treviño M, Oviedo T, Jendritza P, Li SB, Köhr G, De Marco RJ. 2013 Controlled variations in stimulus similarity during learning determine visual discrimination capacity in freely moving mice. Sci. Rep. **3**, 1048. (10.1038/srep01048) 23308341 PMC3541512

[41] Abrahamyan A, Silva LL, Dakin SC, Carandini M, Gardner JL. 2016 Adaptable history biases in human perceptual decisions. Proc. Natl Acad. Sci. USA **113**, E3548–57. (10.1073/pnas.1518786113) 27330086 PMC4922170

[42] Braun A, Urai AE, Donner TH. 2018 Adaptive history biases result from confidence-weighted accumulation of past choices. J. Neurosci. **38**, 2418–2429. (10.1523/JNEUROSCI.2189-17.2017) 29371318 PMC5858589

[43] Urai AE, de Gee JW, Tsetsos K, Donner TH. 2019 Choice history biases subsequent evidence accumulation. eLife **8**, e46331. (10.7554/eLife.46331) 31264959 PMC6606080

[44] Lau B, Glimcher PW. 2005 Dynamic response-by-response models of matching behavior in rhesus monkeys. J. Exp. Anal. Behav. **84**, 555–579. (10.1901/jeab.2005.110-04) 16596980 PMC1389781

[45] Clark A. 2023 The experience machine: how our minds predict and shape reality. New York: Pantheon Books.

[46] Petersch B, Dierkes K. 2022 Gaze-angle dependency of pupil-size measurements in head-mounted eye tracking. Behav. Res. Methods **54**, 763–779. (10.3758/s13428-021-01657-8) 34347276 PMC9046372

[47] de Gee JW, Tsetsos K, Schwabe L, Urai AE, McCormick D, McGinley MJ, Donner TH. 2020 Pupil-linked phasic arousal predicts a reduction of choice bias across species and decision domains. eLife **9**, e54014. (10.7554/eLife.54014) 32543372 PMC7297536

[48] Franzen L, Cabugao A, Grohmann B, Elalouf K, Johnson AP. 2022 Individual pupil size changes as a robust indicator of cognitive familiarity differences. PLoS One **17**, e0262753. (10.1371/journal.pone.0262753) 35061832 PMC8782349

[49] Lawlor J, Zagala A, Jamali S, Boubenec Y. 2023 Pupillary dynamics reflect the impact of temporal expectation on detection strategy. iScience **26**, 106000. (10.1016/j.isci.2023.106000) 36798438 PMC9926307

[50] Lee K, Oh Y, Izawa J, Schweighofer N. 2018 Sensory prediction errors, not performance errors, update memories in visuomotor adaptation. Sci. Rep. **8**, 16483. (10.1038/s41598-018-34598-y) 30405177 PMC6220348

[51] Izawa J, Shadmehr R. 2011 Learning from sensory and reward prediction errors during motor adaptation. PLoS Comput. Biol. **7**, e1002012. (10.1371/journal.pcbi.1002012) 21423711 PMC3053313

[52] Tsay JS, Haith AM, Ivry RB, Kim HE. 2022 Interactions between sensory prediction error and task error during implicit motor learning. PLoS Comput. Biol. **18**, e1010005. (10.1371/journal.pcbi.1010005) 35320276 PMC8979451

[53] Aston-Jones G, Cohen JD. 2005 An integrative theory of locus coeruleus-norepinephrine function: adaptive gain and optimal performance. Annu. Rev. Neurosci. **28**, 403–450. (10.1146/annurev.neuro.28.061604.135709) 16022602

[54] Herz DM *et al*. 2017 Distinct mechanisms mediate speed-accuracy adjustments in cortico-subthalamic networks. eLife **6**, e21481. (10.7554/eLife.21481) 28137358 PMC5287713

[55] Treviño M, De la Torre-Valdovinos B, Manjarrez E. 2016 Noise improves visual motion discrimination via a stochastic resonance-like phenomenon. Front. Hum. Neurosci. **10**, 572. (10.3389/fnhum.2016.00572) 27932960 PMC5120109

[56] Wenzlaff H, Bauer M, Maess B, Heekeren HR. 2011 Neural characterization of the speed-accuracy tradeoff in a perceptual decision-making task. J. Neurosci. **31**, 1254–1266. (10.1523/JNEUROSCI.4000-10.2011) 21273410 PMC6623618

[57] Eberhardt LV, Strauch C, Hartmann TS, Huckauf A. 2022 Increasing pupil size is associated with improved detection performance in the periphery. Atten. Percept. Psychophys. **84**, 138–149. (10.3758/s13414-021-02388-w) 34820766 PMC8795034

[58] Motoyoshi I, Ishii T, Kamachi MG. 2015 Limited attention facilitates coherent motion processing. J. Vis. **15**, 1. (10.1167/15.13.1) 26327254

[59] Ronconi L, Gori S, Ruffino M, Franceschini S, Urbani B, Molteni M, Facoetti A. 2012 Decreased coherent motion discrimination in autism spectrum disorder: the role of attentional zoom-out deficit. PLoS One **7**, e49019. (10.1371/journal.pone.0049019) 23139831 PMC3490913

[60] Binda P, Pereverzeva M, Murray SO. 2014 Pupil size reflects the focus of feature-based attention. J. Neurophysiol. **112**, 3046–3052. (10.1152/jn.00502.2014) 25231615

[61] Unsworth N, Robison MK. 2016 Pupillary correlates of lapses of sustained attention. Cogn. Affect. Behav. Neurosci. **16**, 601–615. (10.3758/s13415-016-0417-4) 27038165

[62] van den Brink RL, Murphy PR, Nieuwenhuis S. 2016 Pupil diameter tracks lapses of attention. PLoS One **11**, e0165274. (10.1371/journal.pone.0165274) 27768778 PMC5074493

[63] Kroell LM, Rolfs M. 2022 Foveal vision anticipates defining features of eye movement targets. eLife **11**, e78106. (10.7554/eLife.78106) 36082940 PMC9581528

[64] Nunnally JC, Knott PD, Duchnowski A, Parker R. 1967 Pupillary response as a general measure of activation. Percept. Psychophys. **2**, 149–155. (10.3758/BF03210310)

[65] Dierkes K, Kassner M, Bulling A. 2019 A fast approach to refraction-aware eye-model fitting and gaze prediction. In Proc. of the 11th ACM Symposium on Eye Tracking Research & Applications, pp. 1–9. New York, NY, USA: Association for Computing Machinery. (10.1145/3314111.3319819)

[66] OSF Home. Pupillary responses to directional uncertainty while intercepting a moving target. See https://osf.io/wbq4d/?view_only=2126de11bea84b38802b781c0dadf35f. 10.1098/rsos.240606 PMC11444787 39359460

[67] Marquez MI, Treviño M. 2024 Data from: Pupillary responses to directional uncertainty while intercepting a moving target. Figshare (10.6084/m9.figshare.c.7461926) PMC11444787 39359460

